# In vitro antibacterial activities of compounds isolated from roots of *Caylusea abyssinica*

**DOI:** 10.1186/s12941-015-0072-6

**Published:** 2015-03-21

**Authors:** Abdissa Edilu, Legesse Adane, Delelegn Woyessa

**Affiliations:** Department of Chemistry, College of Natural Sciences, Jimma University, Jimma, Ethiopia; Department of Biology, College of Natural Sciences, Jimma University, P.O. Box 5140 Jimma, Ethiopia

**Keywords:** Disk diffusion method, β-sitosterol, Secondary metabolite, Stigmasterol

## Abstract

**Background:**

*Caylusea absyssinica,* a plant used as vegetable and for medicinal purposes was selected for in vitro antibacterial evaluation in this study. The main aim of this study was to isolate compounds from the plant roots and evaluate their antibacterial activities on clinical bacterial test strains.

**Methods:**

Compounds from roots of *Caylusea absyssinica* (fresen) were identified based on observed spectral (^1^H-NMR, ^13^C-NMR and IR) data and physical properties (melting point) as well as reported literature. Disk diffusion method was employed to evaluate the antibacterial activities of the isolated compounds on four test bacterial strains namely, *Staphylococcus aureus* (ATCC25903), *Escherichia coli* (ATCC25722), *Pseudomonas aeruginosa (*DSMZ1117) and *Salmonella thyphimurium* (ATCC13311).

**Results:**

Two compounds, CA1 and CA2 were isolated from the methanol crude extract of the roots of *Caylusea absyssinica* (fresen). The compounds were identified as β-sitosterol and stigmasterol, respectively. Evaluation of antibacterial activities revealed that the compounds are active against all the bacterial strains in the experiment, showing inhibition zones ranging from 12 mm-15 mm by CA1 and 11 mm-18 mm by CA2 against the different test strains*.* However, the compounds were less active than the reference drug (Gentamycine), which showed minimum inhibition zone of 21 mm (*Pseudomonas aeruginosa*) and maximum of 28 mm (*Escherichia coli*) inhibition zone.

**Discussion and conclusion:**

The isolation of the compounds is the first report from roots of *Caylusea abyssinica* and could be potential candidates for future antibacterial drug development programs.

**Electronic supplementary material:**

The online version of this article (doi:10.1186/s12941-015-0072-6) contains supplementary material, which is available to authorized users.

## Introduction

Medicinal plants have been used as important drug sources and also to treat various microbial infections [1]. The search for alternative drug sources is currently receiving due attention to tackle the problem of increasing multiple drug resistant microorganisms. *Caylusea Absyssinica* (fresen) is a plant that belongs to the family of Resedaceae. It is an erect herb with height up to 1.5 m tall, and with slightly woody taproot and glabrous stem. This plant mostly grows in open grass land, fields, road sides and rocky areas at 1500–2750 m above sea level. It is distributed in Mediterranean region, and Northern and Eastern Africa. Sudan, Ethiopia, Kenya, Uganda, Rwanda, Burundi, Tanzania and Malawi are some of the Eastern African countries where the plant is found in abundance [2,3].

Previous report indicates the use of *Caylusea absyssinica* as vegetable and medicinal plant [4]. For instance, in Tanzania and Ethiopia, its leaves and stems are eaten alone or as vegetables [2,5]. The plant is also known for its medicinal use by people living in areas where it is growing. Its leaves are used to treat stomachache, skin diseases diabetes mellitus and amoebiasis [6-8]. Similarly, its roots are used to treat abdominal pain impotency and Scabies diarrhea and expel intestinal parasites in humans [9-11]. In Ethiopia, the plant is traditionally used to treat internal diseases, fever, shivering and skin diseases of domestic animals [6,12].

There are some attempts to explore the potentials of crude extracts of *Caylusea abyssinica* against different human diseases [13]. In the report, 80% methanolic extract of leaves of the plant showed antidiabetic and oral glucose tolerance improving actions, particularly at the dose of 200 mg/kg in experimental animals. The report also supported the prevailing traditional claims of the leaves of *Caylusea abyssinica* for management of diabetes mellitus [7].

Isolation of some compounds from different morphological parts of the plant has been reported. For instance, isolation of 3-(3-carboxyphenyl) alanine, (3-carboxyphenyl) glycine,3-(3-carboxy-4-hydroxyphenyl) alanine, and (3-carboxy-4-hydroxyphenyl)-glycine, in low concentration 2-aminoadipic acid, saccharopine [(2*S*, 2′*S*)-*N*^6^-(2-glutaryl)lysine] and some γ-glutamyl peptides have been reported from the leaves of *Caylusea abyssinica* [14]. Phytochemical screening of leaves of 80% methanolic crude extract of *Caylusea abyssinica* revealed the presence of various secondary metabolites such as alkaloids, cardiac glycosides, reducing sugars, steroidal compounds and phenolic compounds, tannins, saponins and flavonoids [13]. However, there is a dearth of study regarding evaluation of compounds from *Caylusea abyssinica* on bacterial pathogens. To the best of our knowledge, there is no report so far on isolation of compounds from the roots of this plant for antibacterial activity tests. This work is, therefore, initiated to isolate compounds from roots of *Caylusea abyssinica* and test their antibacterial activity on four test bacterial strains.

## Materials and methods

### Chemicals and apparatus

General laboratory grade solvents (methanol, acetone, chloroform, petroleum ether and ethyl acetate) were used for gradient extraction and column elution. The materials used for chromatographic analyses were silica gel (60–120 mesh size) and pre-coated TLC (silica gel, UV_254_). A standard antibiotic disc (Gentamycin 10 μg) was used as a reference drug, and Mueller Hinton agar and Nutrient broth were used for preparation of culture media for the antibacterial activity test (experiment). ^1^H-NMR, ^13^C-NMR and DEPT-135 were recorded using Bruker Advance 400 MHz spectrometer. CDCl_3_ was used as a solvent in all NMR spectroscopic analyses. The Infrared (IR) spectra (KBr) data were obtained from Perkin-Elmer BX infrared spectrometer (400–4000 cm^−1^). Melting point apparatus (Griffin) was used for melting point determination.

### Collection and preparation of plant material

The root of *Caylusea absyssinica* was collected in November 2012 from the area surrounding of King Abba Jifar Palace, near Jimma town, Ethiopia. The town’s geographical coordinates are approximately 7°41′N latitude and 36°50′E longitude. Botanical identification of the plant was made by a Botanist, and a specimen with voucher number AE 001 was deposited at the Herbarium of the Department of Biology, Jimma University. The collected plant material was chopped into small pieces and air-dried under shade without exposing it to direct sunlight and the dried plant material was grounded to 0.5 μm sizes.

### Extraction

For preliminary antibacterial activity test, 100 g of the powdered plant material was sequentially extracted with petroleum ether (least polar), chloroform, acetone and methanol (most polar) using maceration technique with continuous shaking (at 25°C for 72 hrs) using a shaker (GSL 400). The extracted matter from each solvent was filtered first using a cotton plug followed by Whatman No 1 filter paper. The filtrates were concentrated using rotary evaporator (Laborota 4000) under reduced pressure. The resulting crude extracts were weighed and stored in refrigerator at 4°C. After comparing the antibacterial activities of the crude extracts of the solvent systems [15], the methanol extract was chosen for further study based on its better inhibitory effect against test strains for chromatographic isolation of its constituents. Then, a bulk of the powdered material (1000 g) was subjected to extraction employing the same procedure (gradient extraction) to afford 28.2 g crude methanol extract.

### Isolation and characterization of compounds

Effective solvent system for column chromatography was selected for elution after carrying out the TLC of the methanol extract in variable combinations of solvents like petroleum ether, ethyl acetate, chloroform and methanol alternatively. Among all combination of solvents, petroleum ether:ethyl acetate combination showed superior resolution of the components of the extract on TLC plate. Therefore, these combinations of solvents of varying polarity were used for elution of column chromatography. About 12 g of methanol crude extract of the roots *Caylusea abyssinica* was subjected to column chromatography (CC) that was packed with silica gel to isolate compounds. A glass column was packed with 120 g silica gel slurry dissolved in petroleum ether. The crude material was adsorbed onto 12 g of dry silica gel. Then the solvent was allowed to evaporate, and the dry sample adsorbed to the silica gel was applied into the column that was already packed with silica gel. The column was then eluted with a mixture of petreoleum ether:ethyl acetate gradually increasing the polarity (i.e., 100:0%, 98:2%, 96:4%, up to 80:20%). A total of 293 fractions each with 20 ml were collected and solvent was removed under reduced pressure using rota vapor (under reduced pressure). The developed spots on TLC plates were visualized under UV light at 254 and 365 nm and then by exposure to iodine chamber. The fractions that showed the same TLC development profiles (color and R_f_) were combined and concentrated to dryness under reduced pressure using rotary evaporator. The structures of the compounds were elucidated based on combined spectral data which include infra red, nuclear magnetic resonance (^1^H-NMR, ^13^C-NMR and DEPT-135) spectra data and melting point values as well as comparison of these data with reported data in literature. All the spectroscopic analyses were carried out at the Department of Chemistry, Addis Ababa University.

### Evaluation of antibacterial activity

*Staphylococcus aureus* (ATCC25903), *Escherichia coli* (ATCC25722), *Pseudomonas aeruginosa* (DSMZ1117) and *Salmonella thyphimurium* (ATCC13311) were clinical test isolates used for antibacterial activity tests. All the test strains were from the Post graduate and Research Laboratory of Biology Department, Jimma University. The antibacterial activity tests were carried out using a standard procedure [16]. All bacterial cultures were first grown on 5% sheep red blood agar Petri plates at 37°C for 24 hrs prior to inoculation onto the nutrient agar. Few colonies (4 to 5) of similar morphology of the respective bacteria were transferred with a sterile inoculating loop to a nutrient broth liquid medium and this liquid culture was incubated until adequate growth of turbidity equivalent to McFarland 0.5 turbidity standard was obtained. The turbidity of the actively growing broth culture was adjusted with sterile saline solution to obtain turbidity optically comparable to that of the 0.5 McFarland standard that was resulted in a suspension containing approximately 1–2 × 10^8^ CFU/ml for the test strains. The respective bacterial culture was streaked onto the Muller-Hinton agar Petri plates using a sterile swab to ensure thorough coverage of the plates and a uniform thick lawn of growth following incubation. Then 6 mm diameter sterile discs (Whatmann No.3 paper) were placed on the surface of the inoculated agar approximately at equal distance of corners in Petri plates in triplicates and 50 mg/ml concentration of the test solutions that were prepared by dissolving 500 mg of isolated compounds in 10 ml of DMSO were also applied onto the discs using micropipette. After addition of test solutions on the discs, they were allowed to diffuse for 5 minutes and the Petri plates were then kept in an incubator at 37°C for 24 hrs. The antibacterial activity was evaluated after 24 hrs by measuring the diameter of zone of growth inhibitions surrounding the discs (in mm) using transparent ruler. In this experiment, Gentamycin (10 μg) and dimethyl sulfoxide (DMSO) were also used as positive and negative controls, respectively.

## Results and discussion

### Antibacterial activity test of compounds **CA1** and **CA2**

Subjecting the compounds to antibacterial activity tests indicated that the antibacterial activities of the isolated compounds were lower than that of the reference drug (Gentamycin 10 μg) against all the bacterial strains used in the experiment and their growth inhibition values were also comparable to each other (Table 1).

**Table 1:** **Inhibition zone (in mm) of the test compounds (compound CA1 and compound CA2) at 50 mg/L and the reference compound (Gentamycin 10** μ**l)**

**S. n** **o.**	**Compounds**	***S. aureus***	***E. coli***	***P. aeruginosa***	***S. thyphimurium***
1	Compound **CA1**	15	13	13	12
2	Compound **CA2**	12	18	11	13
3	DMSO	-	-	-	-
4	Gentamycin	20	28	21	23

As revealed from the current study, the growth inhibitory activities of compound **CA1** are almost the same against the four bacterial strains used in the experiment. i.e., 15 mm, 13 mm, 13 mm and 12 mm for *S. aureus, E. coli, P. aeruginosa* and *S. thyphimurium,* respectively (Table 1). The corresponding activity of Gentamycin against the test strains was found to be 20 mm, 28 mm, 21 mm and 23 mm, respectively. The observed antibacterial activity data of the compound CA1 are in good agreement with corresponding reported antibacterial activity of β-sitosterol against the same bacterial strains. The reported growth inhibitions were 11 mm, 13.5 mm, 8.5 mm and 12 mm against *S. aureus, E. coli, P. aeruginosa* and *S. thyphimurium,* respectively [17]. There are also reports that discuss low/moderate antibacterial activity of β-sitosterol against several bacterial species such as *S. aureus, E. coli*, and *P. aeruginosa* [18-22]. Similarly, the observed antibacterial activities of compound **CA2** against the three test bacterial strains (*S. aureus, P. aeruginosa* and *S. thyphimurium*) were found to be comparable to each other. The corresponding growth inhibitory activities (in mm) were 12, 11 and 13 against *S. aureus, P. aeruginosa* and *S. thyphimurium*, respectively. However, it was found to show relatively superior antibacterial activity (i.e., 18 mm) against *E. coli*. (Table 1). The results of the present study are consistent with previous reports showing low to moderate antibacterial activity of stigmasterol against *S. aureus, E. coli, P. aeruginosa* and *S.* thyphimurium [18,23,24]. For instance, it has been reported that growth inhibition values (in mm) of stigmasterol were 13.5, 14, 9.5 and 13 against *S. aureus, P. aeruginosa* and *S. thyphimurium*, respectively [25].

**Figure 1:**
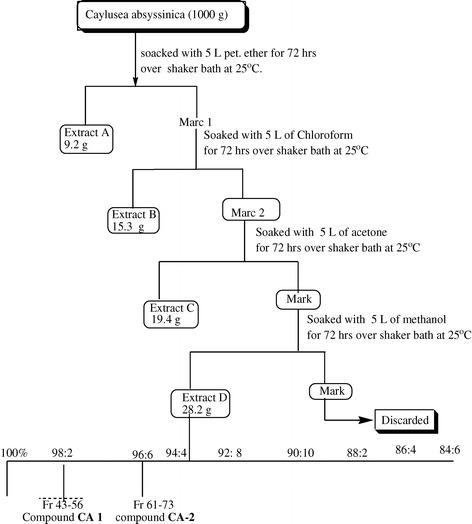
**General procedures followed in the extraction and isolation of compounds from the roots of** ***Caylusea abyssinica.***

Two compounds (compound **CA1** and **CA2**) were isolated from 12 g of methanol crude extract of the roots of *Caylusea abyssinica* using column chromatographic separation*.* Compound **CA1** and compound **CA2** were obtained by combining fractions 43–56 and 61–73, respectively, of the column chromatography (Figure 1). Further characterization of the compounds revealed the compounds to be β-sitosterol [12,13] and stigmasterol [17,25,26], respectively.

### Structural elucidation of the isolated compounds

#### Structural elucidation of compound CA1

Compound **CA1** was obtained as white crystal that was isolated from the combined fractions 43–56 of column chromatographic elution by petroleum ether: ethyl acetate (98:2%). Its R_f_ value was determined to be 0.49 in petroleum ether:ethyl acetate (90:10%) as a solvent system.

**Figure 2:**
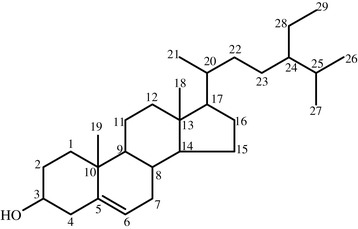
**The proposed chemical structure compound CA1 (i.e.,** **β-sitosterol).**

The IR spectrum of compound **CA1** (Additional file 1) showed a broad band at 3438 cm^−1^ that indicated the presence of -OH group. There is no broad band in the range of 3400–2400 cm^−1^ to be associated with -OH group of carboxylic acids. Moreover, absence of a band at about 1700 cm^−1^ also confirmed that the compound is not a carbonyl compound (or carboxylic acids). Therefore, the band at 2996 cm^−1^ could indicate C-H stretching of alkenes whereas bands at 2853 cm^−1^ and 2938 cm^−1^ could be attributed to C-H stretching of CH_3_ and CH_2_ groups, respectively. The presence of a band at 1174 cm^−1^ could indicate alcoholic C-O stretching. The observed IR spectrum of compound **CA1** agrees with the reported IR spectra of β-sitosterol [12,13]. This claim is further confirmed by NMR spectroscopic and physical property data. The ^1^H-NMR spectrum of compound **CA1** (Additional file 2), showed the presence of six methyl groups corresponding to peaks at δ0.6 (3H, CH_3_-18), 0.82 (3H, CH_3_-29), 0.84 (3H, CH_3_-26), 0.86 (3H, CH_3_-27), 0.88 (3H, CH_3_-21) and 1.02 (3H, CH_3_-19). The peak at δ2.25 (2H, CH_2_-4) was attributed to CH_2_ hydrogen atoms on the fourth carbon. The peak at δ3.53 shows the presence of a proton attached to hydroxyl group bearing carbon (i.e., C-3). The peak at δ5.37 indicates presence of a proton attached to olefinic C-C bond (i.e., C-6) (Figure 2) as reported in [26].

The observed NMR data of compound CA1 and that of reported data of β-sitosterol are given in Table 2. The ^13^C-NMR and DEPT-135 spectra of compound **CA1** (Additional files 3 and 4) revealed that the compound **CA1** possesses a total of 29 carbons atoms. Among these carbons, eleven carbons were found to be CH_2_ carbons whereas nine CH and six CH_3_ carbons. The peaks at δ140.8 and 121.7 can be assigned to highly deshielded carbon atoms of C = C double bond. Thus, peaks values at δ140.8 and 121.7 can be assigned to C-5 and C-6, respectively [17-19]. The peak at δ71.8 can be assigned to carbon atom bearing hydroxyl group (i.e., C-3) [19]. Moreover, the DEPT-135 spectrum confirmed that the peaks at δ140.7, δ36.5 and δ42.3 indicate quaternary carbon atoms. The observed ^13^C-NMR and DEPT-135 spectral data of compound **CA1** (Table 2) are also consistent with the literature reported data β-sitosterol [17,25,26]. The observed melting point value (133–135°C) was also comparable with the reported melting point value of β-sitosterol (i.e. 135–136°C) [27]. Therefore, based on the observed spectral and melting point data, compound **CA1** is most likely β-sitosterol.

**Table 2:** ^**13**^ **C-NMR,** ^**1**^ **H-NMR and DEPT-135 data of CA1 in comparison with reported data of** β**-sitosterol**

**C. no.**	^**13**^ **C-NMR data of compound CA1**	**Reported** ^**13**^ **C-NMR data** **of β-sitosterol** **[** 12 **-** 14 **,** 17 **]**	^**1**^ **H-NMR data of compound CA1**	**Reported** ^**1**^ **H-NMR data of** **β-sitosterol** **[** 12 **-** 14 **,** 17 **]**	**DEPT-135 of compound CA1**	**Nature of C atoms based on DEPT-135**
1	37.25	37.3			37.25	CH_2_
2	31.64	31.6			31.64	CH_2_
3	71.82	71.8	3.54	3.53	71.83	CH
4	42.28	42.2			42.28	CH_2_
5	140.74	140.8			-	C
6	121.74	121.7	5.37	5.37	121.74	CH
7	31.93	31.9			31.93	CH_2_
8	31.93	31.9			31.93	CH
9	50.12	51.2			50.12	CH
10	36.51	36.5			-	C
11	21.08	21.1			21.08	CH_2_
12	39.76	39.8			39.77	CH_2_
13	42.28	42.3			-	C
14	56.76	56.8			56.76	CH
15	24.31	24.3			24.31	CH_2_
16	28.26	28.3			28.26	CH_2_
17	56.04	56.0			56.05	CH
18	11.98	11.9	0.69	0.69	11.99	CH_3_
19	19.41	19.4	1.02	1.02	19.41	CH_3_
20	36.51	36.2			36.56	CH
21	18.78	18.8	0.88	0.88	18.79	CH_3_
22	33.93	33.9			33.94	CH_2_
23	26.04	26.1			26.04	CH_2_
24	45.82	45.9			45.82	CH
25	29.13	29.2			29.13	CH
26	19.83	19.8	0.84	0.84	19.83	CH_3_
27	19.41	19.3	0.86	0.86	19.41	CH_3_
28	23.06	23.1			23.06	CH_2_
29	12.27	12.2	0.82	0.81	12.20	CH_3_

#### Structural elucidation of CA2

Compound CA2 was obtained as a white crystalline solid by combining fractions 61–73 of column chromatographic separation that was eluted by a solvent system of petroleum ether:ethyl acetate (96:4%). Its R_f_ value was determined to be 0.60 in petroleum ether:ethyl acetate (80:20%) solvent system (Table 2).

**Figure 3:**
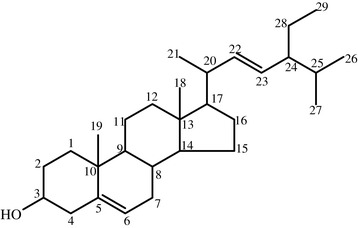
**The chemical structure of compound CA2 (i.e., Stigmasterol).**

The IR (KBr) spectra of compound **CA2** (Additional file 5) showed a broad band at 3424 cm^−1^ which indicates the presence of -OH group. Absence of a broad band in between 3400-2400 cm^−1^ and a strong band at around 1700 cm^−1^ indicated that the compound is not a carboxylic acid (carbonyl compound). Thus, compound **CA2** is most likely an alcohol. The band at 2996 cm^−1^ could indicate C-H stretching of alkenes whereas bands at 2860 cm^−1^ and 2924 cm^−1^ could be attributed to C-H stretching of CH_3_ and CH_2_ groups, respectively. The absorption band at 1657 cm^−1^ is assigned C-C stretching band of C = C double bond. The band at 1465 cm^−1^ could be due to the C-H bending of CH_2_, and the band at 1375 cm^−1^ represents C-H bending of CH_3._ The absorption band at 1055 cm^−1^ corresponds to C-C stretching. The observed IR, ^1^H-NMR and ^13^C-NMR spectral data (Figure 3) and literature reported data suggested that compound **CA2** is most likely stigmasterol [17,25,26].

The ^1^H-NMR spectrum of compound **CA2** (Additional file 6) showed methyl proton peaks at δ0.60 (3H, CH_3_-29), 0.61 (3H, CH_3_-18), 0.72(3H, CH_3_-26), 0.74 (3H, CH_3_-27), 0.76 (3H, CH_3_-21) and 0.93 (3H, CH_3_-19). The peak at δ3.53 indicates proton of hydroxyl group attached to C-3 (i.e., 1H, −OH on C-3). ^1^H-NMR spectrum also showed peaks at δ4.98, 5.06, and 5.26 indicating presence of three protons corresponding to that of a trisubstituted and a disubstituted C = C bonds (Table 3).

**Table 3:** **The observed** ^**13**^ **C-NMR, DEPT-135 and** ^**1**^ **H-NMR data of compound CA2, and the reported** ^**13**^ **C-NMR and** ^**1**^ **H-NMR data of stigmasterol**

**C. n** **o** **.**	^**13**^ **C-NMR data of compound CA2**	**The reported** ^**13**^ **C-NMR data of stigmasterol** **[** 12 **-** 14 **,** 17 **]**	**DEPT-135 data of compound CA2**	^**1**^ **H-NMR data of compound CA2**	**The reported** ^**1**^ **H-NMR data of stigmasterol** **[** 12 **-** 14 **,** 17 **]**	**Dept-135 based nature of the carbon**
1	37.2	37.3	37.2			CH_2_
2	31.6	31.6	31.6			CH_2_
3	71.8	71.8	71.8	3.44	3.45	CH
4	42.2	42.3	42.3			CH_2_
5	140.7	140.8	-			C
6	121.7	121.7	121.7	5.26	5.33	CH
7	31.9	31.9	31.9			CH_2_
8	31.9	31.9	31.9			CH
9	50.1	51.2	50.1			CH
10	36.5	36.5	-			C
11	21.0	21.1	21.0			CH_2_
12	39.7	39.7	39.7			CH_2_
13	42.3	42.3	-			C
14	56.8	57.9	56.7			CH
15	24.3	24.4	24.4			CH_2_
16	28.9	28.4	28.2			CH_2_
17	55.9	56.1	55.9			CH
18	12.2	11	12.2	0.61	0.68	CH_3_
19	19.4	21.2	19.4	0.93	1.02	CH_3_
20	40.5	40.5	40.5			CH
21	21.1	21.2	21.1	0.76	1.01	CH_3_
22	138.3	138.3	138.3	5.06	5.12	CH
23	129.2	129.3	129.2	4.96	4.98	CH
24	51.2	51.2	51.2			CH
25	33.9	31.9	33.7			CH
26	19.0	19.0	19.0	0.72	0.86	CH_3_
27	21.2	21.2	21.2	0.74	0.71	CH_3_
28	25.4	25.4	25.4			CH_2_
29	12.0	12.1	11.8	0.60	0.78	CH_3_

The observed ^13^C-NMR (and DEPT-135) data were also found to be consistent with that of stigmasterol (Table 3). ^13^C-NMR and Dept-135 spectra of compound **CA2** (Additional files 7 and 8) showed presence of a total of 29 carbons atoms in the structure. The peaks were related to six methyl, nine methylene, eleven methane and three quaternary carbon atoms. The peaks at δ140.7, 121.7, 138.3 and 129.2 in the ^13^C-NMR spectrum are assigned to C-5, C-6_,_ C-22 and C-23 of C = C double bonds, respectively. The peaks at δ140.7 and 121.7 are assigned to C = C double bond carbons (C-5 and C-6, respectively) in the cyclic structure of the compound. Reports showed that the existence of unsaturation between C-5 and C-6 introduces easily recognizable signals at δ141.2 ± 0.8 and 121 ± 0.4 [14]. The peaks at δ138.3 and 129.2 assignable to the external C = C double bond carbon atoms, and the peak at δ71.8 is associated to the β hydroxyl carbon of C-3 [13,14]. The DEPT-135 spectrum of compound CA2 also confirmed that the peaks at δ140.7, δ36.5 and δ42.3 (Table 2) indicate quaternary carbon atoms. Thus, based on the above spectral data, and also comparing with literature reports, compound CA2 is found to be identical with stigmasterol (Figure 3). The observed mp value (i.e., 173–176°C) was also found to be comparable with the reported mp value (i.e., 174–176°C) [28]. This is the first report on isolation of stigmasterol from *Caylusea abyssinica* as well as its family.

## Conclusions

In vitro antibacterial activity test results against the four bacterial strains (*S. aureus, E. coli, P. aeruginosa and S. thyphimurium*) used in the experiment, both compounds (CA1 and CA2) showed lower (but moderate) antibacterial activities than the reference drug (Gentamycin). Though lower than that of the reference drug, the observed antibacterial activities of the isolated compounds could give insight about the potentials the compounds as lead compound in development of antibacterial drugs. However, further tests are recommended on large number of bacterial strains to decide their potential as candidates in development of antibacterial drugs.

## Additional files

Additional file 1:
**IR spectrum of compound CA1.**
Additional file 2:
**The** ^**1**^ **H-NMR spectrum of compound CA1.**
Additional file 3:
**The** ^**13**^ **C-NMR Spectra of compound CA 1.**
Additional file 4:
**The DEPT-135 spectrum of compound CA 1.**
Additional file 5:
**The IR spectrum of compound CA2.**
Additional file 6:
**The** ^**1**^ **H-NMR spectrum of compound CA2.**
Additional file 7:
**The** ^**13**^ **C-NMR spectrum of compound CA2.**
Additional file 8:
**The DEPT-135 spectrum of compound CA2.**
