# Supplementary figures and images for: In vitro antibacterial activities of compounds isolated from roots of Caylusea abyssinica
Source: Ann Clin Microbiol Antimicrob. 2015 Mar 21;14:15. doi: 10.1186/s12941-015-0072-6 (PMC4379615; doi:10.1186/s12941-015-0072-6)

Supplementary material 1. IR spectrum of compound **CA1**

**
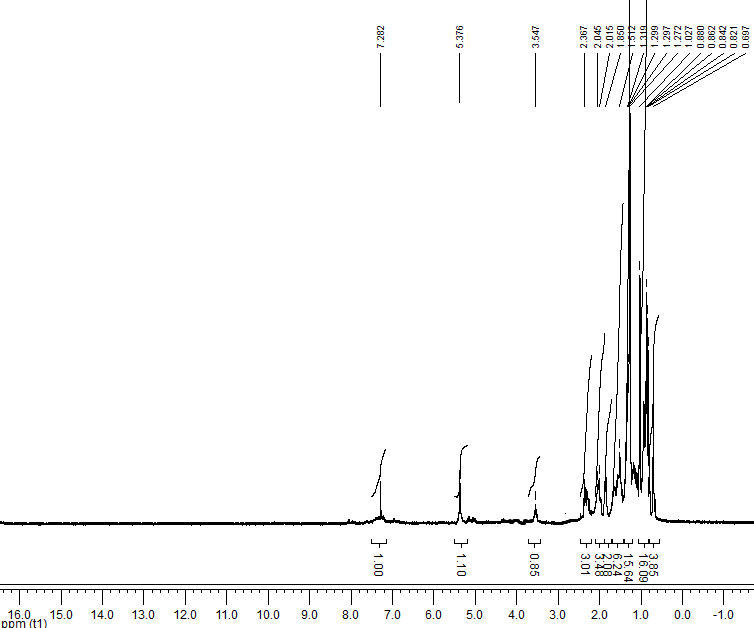
**

Supplement: Additional file 2: — The 1 H-NMR spectrum of compound CA1. [file 12941_2015_72_MOESM2_ESM.doc]

**Supplementary material 3**. The 13C-NMR Spectra of compound **CA 1**


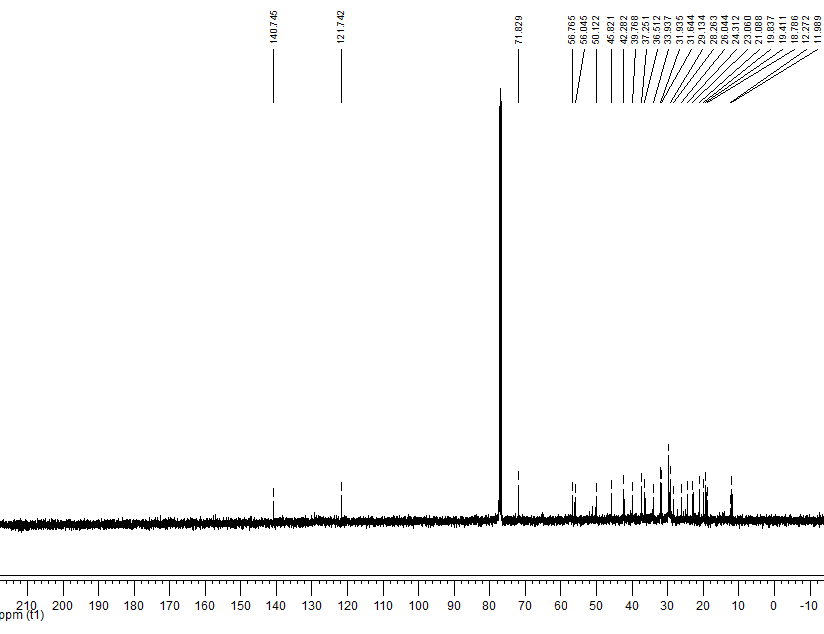

Supplement: Additional file 3: — The 13 C-NMR Spectra of compound CA 1. [file 12941_2015_72_MOESM3_ESM.doc]

**Supplementary material 4**. The DEPT-135 spectrum of compound **CA 1**


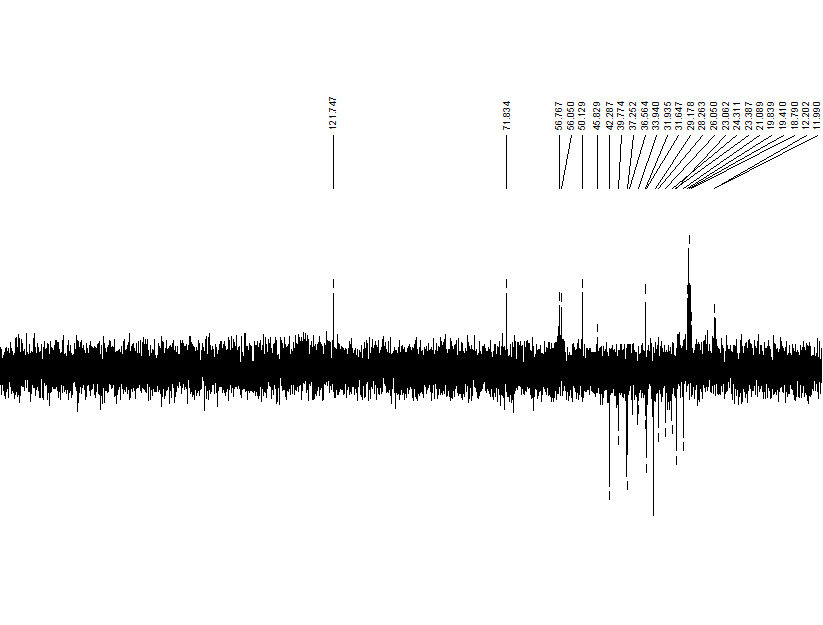

Supplement: Additional file 4: — The DEPT-135 spectrum of compound CA 1. [file 12941_2015_72_MOESM4_ESM.doc]

**Supplementary material 6.** The 1H-NMR spectrum of compound CA2


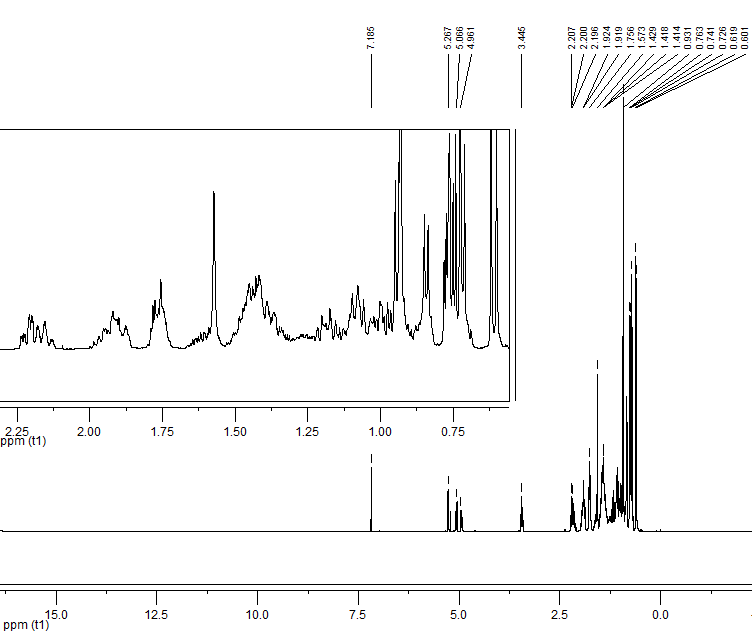

Supplement: Additional file 6: — The 1 H-NMR spectrum of compound CA2. [file 12941_2015_72_MOESM6_ESM.doc]

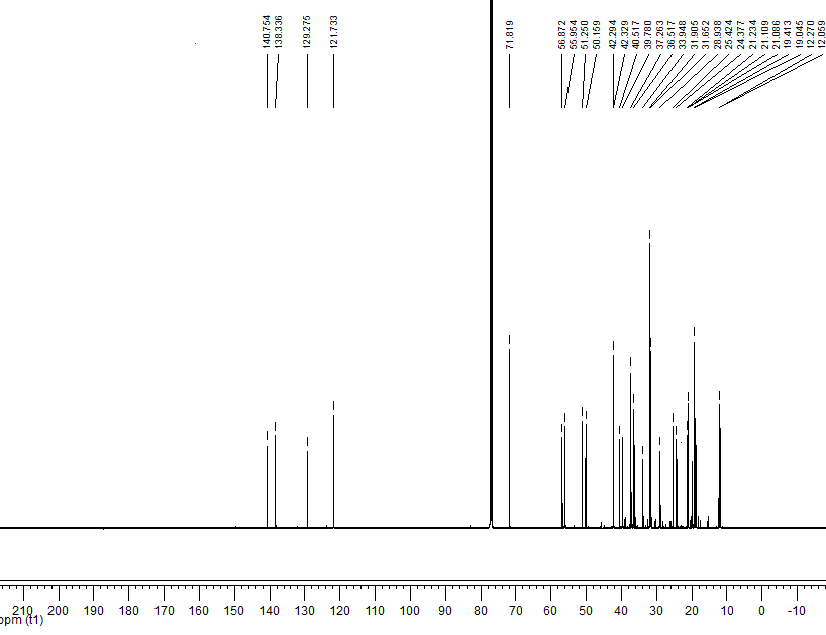
**Supplementary material** 7. The 13C-NMR spectrum of compound **CA2**

Supplement: Additional file 7: — The 13 C-NMR spectrum of compound CA2. [file 12941_2015_72_MOESM7_ESM.doc]

**Supplementary material 8.** TheDEPT-135spectrum of compound **CA2**

**
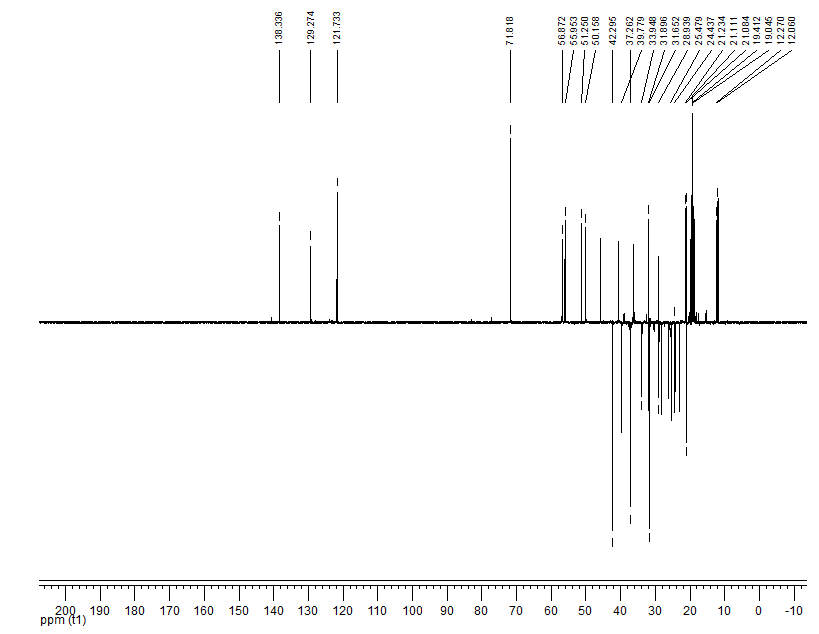
**

Supplement: Additional file 8: — The DEPT-135 spectrum of compound CA2. [file 12941_2015_72_MOESM8_ESM.doc]
