# Supplementary material for: In vitro antibacterial activities of compounds isolated from roots of Caylusea abyssinica
Source: Ann Clin Microbiol Antimicrob. 2015 Mar 21;14:15. doi: 10.1186/s12941-015-0072-6 (PMC4379615; doi:10.1186/s12941-015-0072-6)
Supplement: Additional file 5: — The IR spectrum of compound CA2. [file 12941_2015_72_MOESM5_ESM.doc]

**Supplementary material 5**: The IR spectrum of compound **CA2**
